# Short-form adaptive measure of financial toxicity from the Economic Strain and Resilience in Cancer (ENRICh) study: Derivation using modern psychometric techniques

**DOI:** 10.1371/journal.pone.0272804

**Published:** 2022-08-25

**Authors:** Cai Xu, Grace L. Smith, Ying-Shiuan Chen, Cristina M. Checka, Sharon H. Giordano, Kelsey Kaiser, Lisa M. Lowenstein, Hilary Ma, Tito R. Mendoza, Susan K. Peterson, Ya-Chen T. Shih, Sanjay Shete, Chad Tang, Robert J. Volk, Chris Sidey-Gibbons

**Affiliations:** 1 Symptom Research CAO, The University of Texas MD Anderson Cancer Center, Houston, Texas, United States of America; 2 GI Radiation Oncology, The University of Texas MD Anderson Cancer Center, Houston, Texas, United States of America; 3 Radiation Oncology Clinical Research, The University of Texas MD Anderson Cancer Center, Houston, Texas, United States of America; 4 Breast Surgical Oncology, The University of Texas MD Anderson Cancer Center, Houston, Texas, United States of America; 5 Health Services Research, The University of Texas MD Anderson Cancer Center, Houston, Texas, United States of America; 6 General Medical Oncology, The University of Texas MD Anderson Cancer Center, Houston, Texas, United States of America; 7 Behavioral Science, The University of Texas MD Anderson Cancer Center, Houston, Texas, United States of America; 8 Biostatistics, The University of Texas MD Anderson Cancer Center, Houston, Texas, United States of America; 9 Radiation Oncology Department, The University of Texas MD Anderson Cancer Center, Houston, Texas, United States of America; University of Pennsylvania, UNITED STATES

## Abstract

**Objectives:**

This study sought to evaluate advanced psychometric properties of the 15-item Economic Strain and Resilience in Cancer (ENRICh) measure of financial toxicity for cancer patients.

**Methods:**

We surveyed 515 cancer patients in the greater Houston metropolitan area using ENRICh from March 2019 to March 2020. We conducted a series of factor analyses alongside parametric and non-parametric item response theory (IRT) assessments using Mokken analysis and the graded response model (GRM). We utilized parameters derived from the GRM to run a simulated computerized adaptive test (CAT) assessment.

**Results:**

Among participants, mean age was 58.49 years and 278 (54%) were female. The initial round factor analysis results suggested a one-factor scale structure. Negligible levels of differential item functioning (DIF) were evident between eight items. Three items were removed due to local interdependence (Q3>+0.4). The original 11-point numerical rating scale did not function well, and a new 3-point scoring system was implemented. The final 12-item ENRICh had acceptable fit to the GRM (*p*<0.001; TLI = 0.94; CFI = 0.95; RMSEA = 0.09; RMSR = 0.06) as well as good scalability and dimensionality. We observed high correlation between CAT version scores and the 12-item measure (r = 0.98). During CAT, items 2 (money you owe) and 4 (stress level about finances) were most frequently administered, followed by items 1 (money in savings) and 5 (ability to pay bills). Scores from these four items alone were strongly correlated with that of the 12-item ENRICh (r = 0.96).

**Conclusion:**

These CAT and 4-item versions provide options for quick screening in clinical practice and low-burden assessment in research.

## Introduction

The use of advanced treatments and medical care facilities to diagnose and treat cancer improved outcomes and prolonged the life of patients [1,2]. However, the cost of these advanced treatments are increasing and patients themselves are paying a greater proportion of the costs of treatment [3,4]. Approximately 48% -73% of cancer survivors experience adverse financial effects of cancer treatment whether directly from costs of treatment or indirectly from lost income or ability to work [5]. In the United States, the greatest financial burden of cancer treatment is experienced by adults aged between 18 and 64 years [6].

In this study, we use the term financial toxicity to describe the negative effects on cancer patients’ subjective and material experience resulting from the cost of cancer care [7,8]. The potential consequences of such adverse impact may manifest as material losses, psychological distress, and maladaptive coping strategies [8]. Therefore, financial toxicity should be assessed within material, psychological, and behavioral domains [6,9].

Though multi-level strategies to prevent and mitigate financial toxicity have been proposed [10], the effectiveness of such strategies is dependent on accurately identifying individuals at high risk of financial toxicity and measuring the severity of financial toxicity. Currently, there are three validated financial hardship patient-reported outcome measures (PROMs) of financial toxicity. The COmprehensive Score for financial Toxicity-Functional Assessment of Chronic Illness Therapy (COST-FACIT) tool with 12 items measures general financial toxicity [11,12]. The InCharge Financial Distress/Financial Well-Being scale with 8 items focuses on psychological distress-based financial hardship [13]. Our group developed the 15-item Economic Strain and Resilience in Cancer (ENRICh) measure to comprehensively encompass material, psychological, and behavioral coping dimensions(see S1 Table) [14,15]. The scoring range of 0 to 10 for items indicates the none and the highest burden, respectively. The previous studies on patients with stage I-IV cancer show the overall mean of financial hardship score measured by ENRICh was 3.56 (sd = 2.64) [15], and the mean score for socioeconomically disadvantaged patients was 2.3 times higher [14]. However, to date, the advanced psychometric properties of this measure and its potential suitability for brief computational measurement using computerized adaptive testing (CAT) has not been assessed.

Computerized adaptive testing refers to the process that the computer automatically administers an item from the item bank most relevant to the questionnaire-taker based on his/her response to the last item [16]. Previous research has demonstrated the CAT approach successfully shortens the length of fixed scale as much high as 82% by reducing the number of items to be administered [17–20], which makes more efficient and personalized PROM assessment possible [17]. Computerized adaptive testing is made possible by the application of item response theory, a probabilistic framework that can be used to assess the advanced measurement properties of questionnaires [21,22], administer the personalized measure to questionnaire—taker, and facilitate the development of short and effective version of PRO measure by detecting items with most information. The methodology is widely used in educational assessment and is beginning to be used more in health assessment [17]. The previous studies on IRT-based CAT tools delivery have shown CAT algorithms’ promising application prospects and possibilities through the construction of goal-oriented implementation platforms [23].

In this study, we aimed to apply advanced psychometric methods to data collected using the ENRICh to assess the suitability of the scale for CAT-based assessment. In doing so, we will evaluate the measure’s advanced psychometric properties and assess the potential to create a shorter version of the measure to screen cancer patients at high risk of financial toxicity. The resulting shorter version of the measure will reduce the respondent burden for patients with cancer and can be used with confidence in clinical practice.

## Methods

### Participants

As a part of the Economic Strain and Resilience in Cancer (ENRICh) study, a total of 515 English-speaking participants, aged 18 and older receiving ambulatory oncology care in the greater Houston metropolitan area, were surveyed from participating medical, surgical, or radiation oncology clinics between March 2019 and March 2020. This study cohort was a subgroup of a parent study of 628 patients. Overall response rate was 69.1%. Patients underwent this survey in an institutional review board approved protocol from the MD Anderson Cancer Center (IRB 2016–0391). Patients provided informed consent by reading a consent statement provided before the survey. There was a waiver of written consent. No minors were in the study.

Besides the financial stress assessed using 15-item ENRICh measure, we also collected their basic clinical and socioeconomic information(see S2 Table for details).The mean score of the patients’ age was 58.49(sd = 12.31), among which, 346(67%) were younger adults (≤65), 278(54%) were female, and 335(65%) were White, 505(98%) had more than 1 types of insurance, and 243(47%) were non-metastatic at diagnosis.

### Financial toxicity assessment

The 15-item ENRICh measure is a newly designed PROM to capture respondents’ overall financial toxicity comprised from the dimensions of direct material burden, psychological burden, and depletion of coping resources [14,24], resulting from cancer and its treatment.

Each item is scored using an 11-point numerical rating scale with higher scores indicating increased financial burden at any point in the cancer trajectory. The median time from cancer diagnosis to survey was 267 days (IQR, 122.0, 535.5). It has acceptable reliability and validity for assessing cancer-related financial burden [14]. Consistent with the iterative nature of validation, we sought to assess the scale’s advanced psychometric properties and suitability for CAT.

### Analysis strategies

#### Missing data

Demographic information (e.g., gender, age, race) and 15 items of the ENRICh were incorporated into the series of analyses. As less than 3% of data was missing for each item, a multiple imputation approach was employed to handle the missing data by using predictive mean matching for numerical variables to reduce bias [25]. We used imputed dataset of 515 patients to conduct the following advanced psychometric analyses-IRT and CAT. Imputation was necessary for Mokken analysis. Of note, this large sample size is likely to cause type I error resulting in a significant *p*-value in the chi-squared (χ^^2^) test [26].

#### IRT analysis and CAT simulation

We first assessed the scale data’s eligibility for conducting the IRT analysis, that is, whether it had met the specific assumptions of unidimensionality, scalability, and local independence of items, which determined whether item parameters could be calibrated successfully to further build item bank for subsequent CAT simulation conducting. During the assessment process, where needed, appropriate and necessary modifications were made to ensure the rigorous assumptions had met. We then conducted three CAT simulations at varied SEs of 0.32,0.45,0.55 and compared their performances. The specific principles and mechanisms with details for IRT analysis can be referred from somewhere else [17]. The detailed analysis processes for IRT and CAT in this study were summarized in S1 Text.

### Software

We conducted all the IRT analyses with packages of “lavaan”, “mokken”, “mirt”, “lordif”. We simulated CAT using code derived from the Firestar package [27], and agreement with the fixed-length ENRICh tool using the "BlandAltmanLeh" package. All analyses were completed within the R Statistical software Version 4.1.1.

## Results

### Unidimensionality test

Results of initial confirmatory factor analysis (CFA) in Table 1 showed that although all the factor loadings of included 15 items were greater than the threshold of 0.3, the fit statistics indicated a poor confirmatory model fit (χ^^2^, *p*<0.001; TLI = 0.74; CFI = 0.78; RMSEA = 0.15; RMSR = 0.08). Therefore, we conducted exploratory factor analysis (EFA) to further investigate the dimensional structure of the ENRICh measure. Parallel analysis suggested the existence of two components, however, factor analysis revealed only one factor with eigenvalue value greater than 1. As the second component was very weak with an eigenvalue of 1.50, and one dominant factor with an eigenvalue of 7.34 was apparent, we chose to proceed with a single factor structure for the remainder of the analyses.

**Table 1:** Item descriptive statistics and factor loadings for the ENRICh.

Item	Mean	SD	Factor Loading^a^
Item 1	4.18	3.88	0.79 (0.77)
Item 2	2.91	3.74	0.77 (0.74)
Item 3	4.33	3.86	0.65 (0.64)
Item 4	4.24	3.84	0.86 (0.81)
Item 5	2.70	3.71	0.79 (0.71)
Item 6	1.92	3.23	0.69
Item 7	3.32	4.20	0.61 (0.57)
Item 8	3.59	3.65	0.68 (0.55)
Item 9	4.10	3.85	0.80 (0.75)
Item 10	3.41	3.90	0.74 (0.72)
Item 11	2.88	3.76	0.60 (0.58)
Item 12	1.59	3.22	0.45 (0.42)
Item 13	3.35	3.78	0.58
Item 14	2.17	3.49	0.52 (0.46)
Item 15	1.32	2.92	0.43

^a^ Final round of analysis without items 6, 13, and 15 yielded results in parentheses.

### Scalability of items

As polytomous items had more than 10 response categories each, Mokken analysis was inapplicable to help identify the unidimensional structure found from EFA results, or to evaluate the item homogeneity to test scalability assumption.

### Differential item functioning

**Fig 1:**
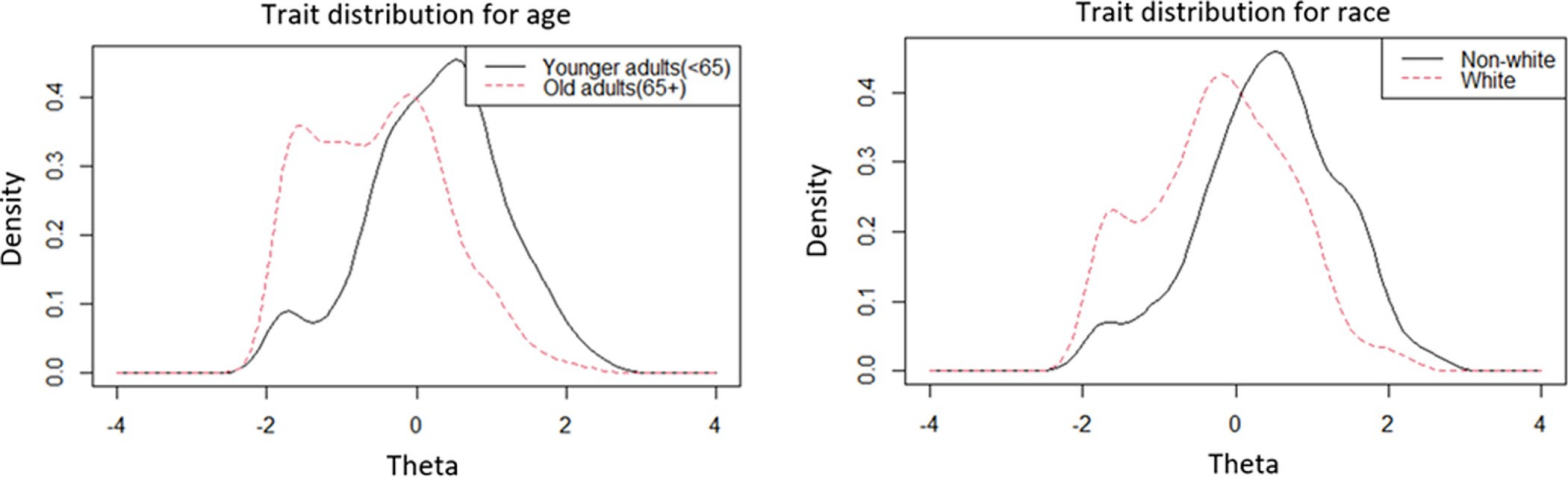
Trait distributions for age and race.

Table 2 of DIF results showed eight uniform DIF items found for age group (2) and race group (6), and no DIF issue within gender. Slight differences were observed in trait distribution for age and race groups in Fig 1. Younger adults (<65) and non-white groups were likely to experience more severe financial hardship relating to cancer treatment than their respective counterparts. The magnitude of all DIF items was small with Pseudo *R*^2^ ranging from 0.004 to 0.03, therefore, their impact was considered negligible.

**Table 2:** Comparison results of significant DIF among younger vs old adults and non-white vs white patients using ordinal logistic regression models.

Variable	Number of categories	Test for uniform DIF^b^		Test for non-uniform DIF^c^	Test for overall DIF^d^
		*R* ^*^2*^	β	*R* ^^2^	*R* ^^2^
**Detected DIF item for Age**					
Item3 (spending on medical bills)	9	0.01*^a^	0.04	0.01	0.01*
Item 7 (ability to work)	4	0.03*	0.09	0.01	0.04*
**Detected DIF item for Race**					
Item 2 (money you owe)	9	0.01*	0.03	<0.001	0.01*
Item 3 (spending on medical bills)	11	0.004*	0.06	0.003	0.01*
Item 5 (ability to pay bills)	10	0.01*	0.02	<0.001	0.02*
Item 6 (ability to pay for food)	7	0.03*	0.02	0.002	0.03*
Item 10 (using your savings)	11	0.01*	0.08	0.002	0.01*
Item 15 (help from communities)	5	0.03*	0.12	<0.001	0.03*

^a^ * denotes *p* value <0.01.

^b^ model 1 versus model 2.

^c^ model 2 versus model 3.

^d^ model 1 versus model 3.

### IRT GRM results

All the F1 scores were greater than 0.60 indicating adequate loading (see Table 3). There 14 items had discrimination(a) higher than the threshold of 1.35 except item 15(a = 1.26) [28].

**Table 3:** Discrimination and difficulty parameter estimates for the ENRICh measure.

Item	a^a^	b1^a^	b2^a^	b3	b4	b5	b6	b7	b8	b9	b10	Factor 1^a^
Item 1	2.85 (2.81)	-0.51 (-0.48)	-0.33 (1.13)	-0.15	0.02	0.14	0.36	0.52	0.72	0.91	1.15	0.86 (0.86)
Item 2	2.95 (2.89)	0.06 (0.07)	0.17 (1.33)	0.36	0.49	0.55	0.74	0.85	0.98	1.21	1.34	0.87 (0.86)
Item 3	1.88 (1.92)	-0.80 (-0.75)	-0.51 (1.26)	-0.27	-0.03	0.13	0.41	0.53	0.69	0.93	1.27	0.74 (0.75)
Item 4	4.05 (3.53)	-0.55 (-0.54)	-0.34 (1.08)	-0.14	0.02	0.16	0.39	0.48	0.65	0.86	1.08	0.92 (0.90)
Item 5	3.44 (2.73)	0.14 (0.17)	0.31 (1.36)	0.42	0.52	0.60	0.74	0.85	0.99	1.19	1.31	0.70 (0.85)
Item 6	2.76	0.45	0.57	0.72	0.86	0.95	1.20	1.30	1.37	1.52	1.65	0.85
Item 7	1.63 (1.57)	0.08 (0.10)	0.23 (1.17)	0.37	0.45	0.53	0.71	0.77	0.87	1.00	1.16	0.69 (0.68)
Item 8	1.76 (1.45)	-0.53 (-0.56)	-0.26 (1.90)	0.02	0.21	0.42	0.72	0.90	1.09	1.41	1.72	0.72 (0.65)
Item 9	2.88 (2.64)	-0.60 (-0.59)	-0.36 (1.06)	-0.12	0.15	0.30	0.56	0.63	0.75	0.92	1.07	0.86 (0.84)
Item 10	2.44 (2.43)	-0.25 (-0.20)	-0.02 (1.20)	0.15	0.29	0.39	0.55	0.70	0.85	1.05	1.21	0.82 (0.82)
Item 11	1.64 (1.65)	0.00 (0.04)	0.22 (1.57)	0.39	0.57	0.69	0.92	1.03	1.15	1.45	1.60	0.69 (0.70)
Item 12	1.40 (1.26)	1.08 (1.14)	1.22 (2.40)	1.29	1.37	1.48	1.66	1.74	1.82	2.11	2.24	0.63 (0.59)
Item 13	1.38	-0.29	-0.10	0.18	0.38	0.51	0.90	1.00	1.24	1.49	1.75	0.63
Item 14	1.41 (1.25)	0.50 (0.54)	0.67 (2.06)	0.87	1.02	1.12	1.39	1.55	1.66	1.77	1.92	0.64 (0.59)
Item 15	1.26	1.25	1.42	1.51	1.59	1.72	2.05	2.17	2.35	2.43	2.53	0.60

^a^ Results for the final round analysis including 12 items with recoded 3 response categories each are in parentheses.

**Fig 2:**
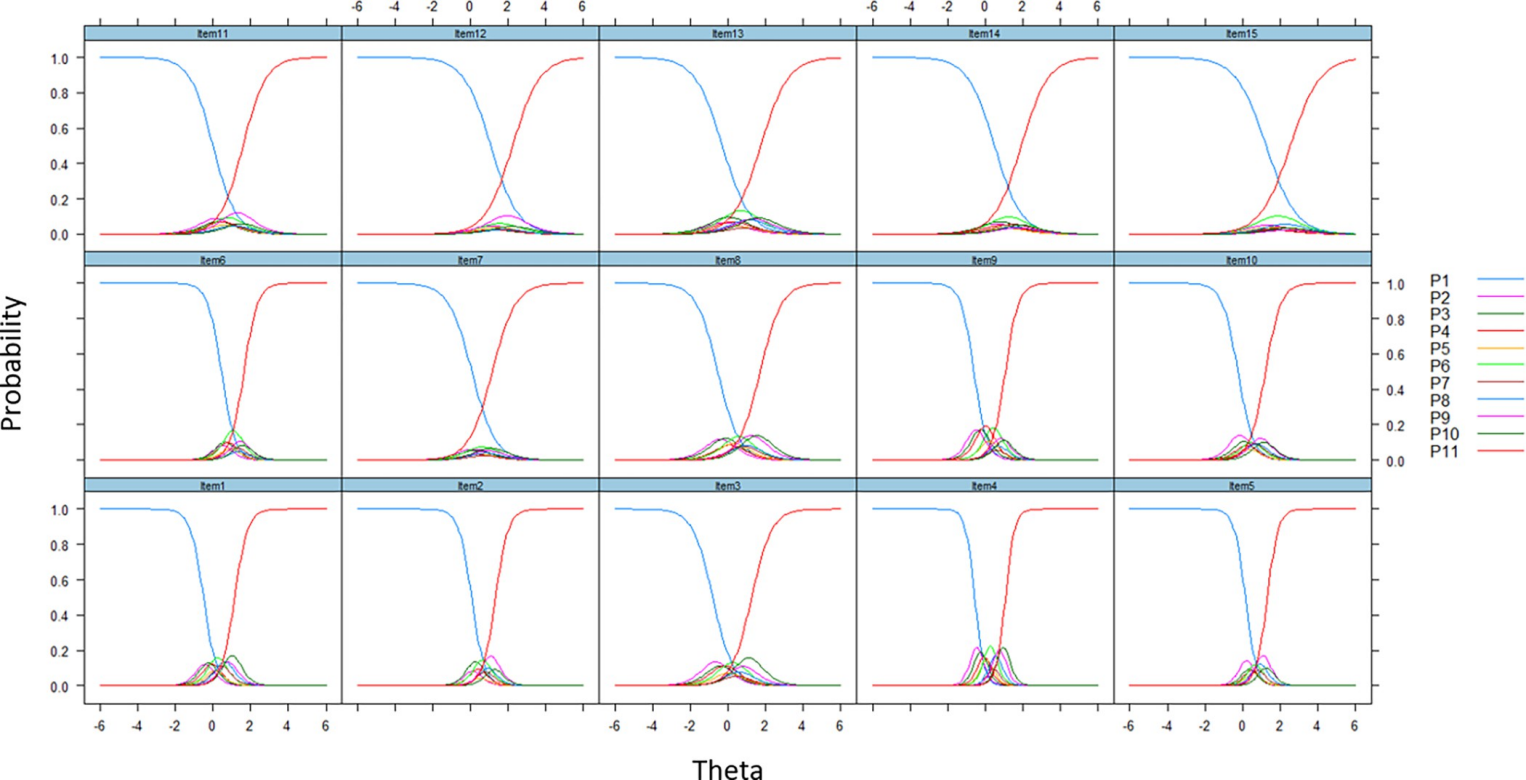
Disordered thresholds for initial analysis on 15 items with 11 response categories each displayed as 0-1-2-3-4-5-6-7-8-9-10.

Furthermore, the item characteristic curve showed that all the 15 items with disordered threshold issues (see Fig 2). The histogram of each item verified this uneven distribution as well. Therefore, we addressed this issue by recoding the thresholds for all items.

### Local independence of items test

Local independence of item assumption was reasonable for most items. However, item residual correlations among items 5 (ability to pay all of your bills) and 6 (ability to pay for food) (Q3 = +0.49), items 8 (ability to contribute to your normal household responsibilities and daily chores) and 13 (having someone to help with your normal household responsibilities and daily chores)(Q3 = +0.48), and items 14 (having someone to help care for the people who normally depend on you) and 15 (having help from community resources)(Q3 = +0.40) were higher than recommend a cutoff of +0.2. As lower information was provided by items 6, 13, and 15 compared with items 5, 8 and 14, respectively based on their item information curve, therefore, items 6, 13, and 15 were eliminated from the final round analysis below.

### Analysis results of final round of IRT assumption test

After appropriate item modification and rescoring moves, the remaining 12 items were reanalyzed. Sufficient factor loadings were revealed and are demonstrated in parentheses in Table 1. The fit of confirmatory model was improved (χ^^2^, *p*<0.001; TLI = 0.87; CFI = 0.89; RMSEA = 0.10; RMSR = 0.06). The parallel analysis screen plot showed there was only one component, and ASIP of Mokken analysis verified this finding by showing that all the 12 items scaled onto a single scale. In addition, results of Mokken analysis in Table 4 indicated that both each item and whole scale had achieved sufficient scalability as all Loevinger’s H coefficients were greater than 0.30.

**Table 4:** Loevinger’s coefficient for scalability assumption test from Mokken analysis.

Item	Mean	ItemH (H_i_)^a^	Stand Error	Dimensionality^b^
Item 1	4.18	0.55	0.03	1
Item 2	2.91	0.54	0.02	1
Item 3	4.33	0.49	0.03	1
Item 4	4.24	0.59	0.02	1
Item 5	2.70	0.54	0.03	1
Item 7	3.32	0.46	0.03	1
Item 8	3.59	0.45	0.03	1
Item 9	4.10	0.56	0.02	1
Item 10	3.41	0.53	0.03	1
Item 11	2.88	0.43	0.03	1
Item 12	1.59	0.40	0.04	1
Item 14	2.17	0.40	0.03	1

^a^Scale H for final round analysis with 12 items are 0.50(0.02).

^b^Results for the first round of Mokken analysis is not available as Mokken can’t handle up to 10 categories for included items.

**Fig 3:**
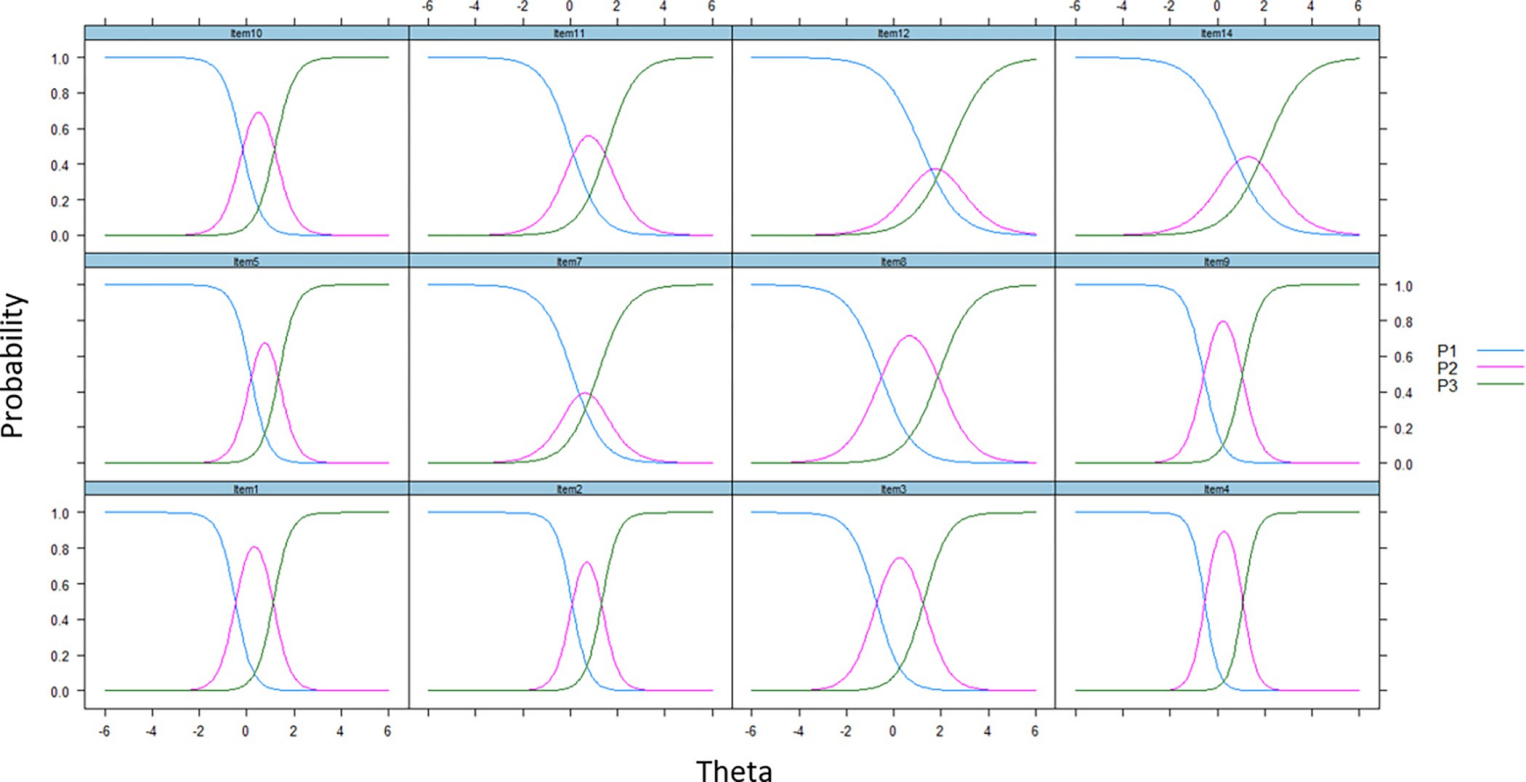
Recoded thresholds for final analysis on 12 items with 3 response categories each displayed as 0-1-1-1-1-1-1-1-1-1-2.

**Fig 4:**
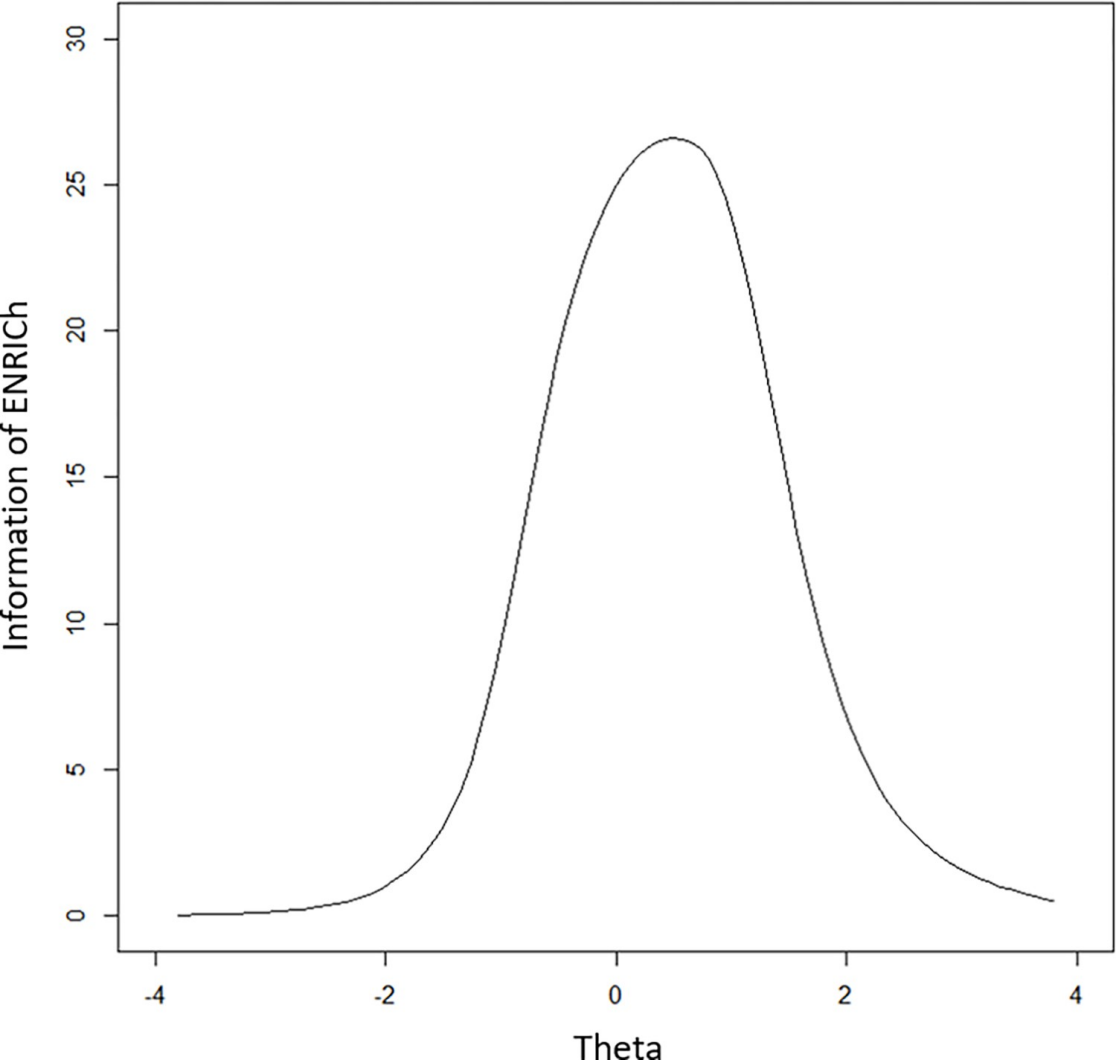
Test information curve of the ENRICh with 12 items.

The results of reanalyzed IRT GRM are presented in Table 3 in parentheses. The number of thresholds for difficulties (*b*) reduced to 2 from 10. The collapsed thresholds displayed in Fig 3 demonstrated the disordered items issue had been resolved. The test information curve (Fig 4) showed that the most test information was concentrated on the theta(θ) of 1. The mean for factor scores of the whole scale was 0.0005 (sd = 0.95). The GRM achieved acceptable model fit based on the fit (TLI = 0.94; CFI = 0.95; RMSEA = 0.09; RMSR = 0.06). The revised ENRICh measure showed better psychometric properties than before (see S3 Table).

### Results of CAT simulation

**Fig 5:**
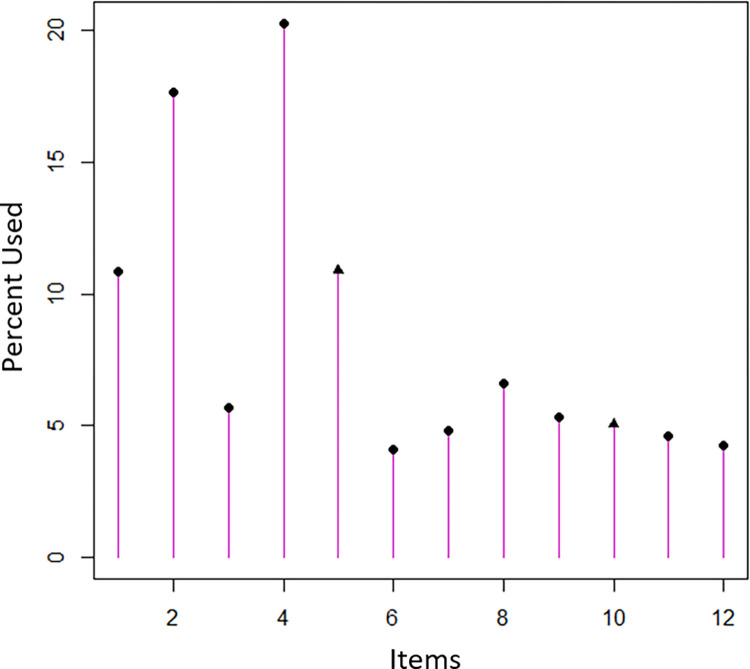
Frequency of items used in the ENRICh CAT simulation.

The results of three CAT simulations with varied stopping rules (SEs at 0.32, 0.45, and 0.55) are presented in Table 5. The lowest average number of items used during simulations was 2, whereas, the correlation of thetas derived from the CAT simulation and that from the fixed 12-item measure were as high as 0.98 when SE was set to 0.32.Items 2 (money you owe) and 4 (your stress level about finances) with most information were most frequently used during the CAT simulation (see Fig 5), followed by items 1 (money in your savings) and 5 (ability to pay bills). The factor scores obtained from items 2 and 4 only were closely correlated to those derived from the fixed 12-item measure (r = 0.85, *p*<0.001). After adding items 1 and 5, the factor scores of the 4-item ENRICh was much more closely associated with that of fixed 12-item measure (r = 0.96, *p*<0.001), as they provided 97.04% of item information at the theta range of (-2,+2) in Table 6.

**Table 5:** Results of three ENRICh CAT simulations with varied SEs.

	SE (0.32)	SE (0.45)	SE (0.55)
Alpha(α)	.90	.80	.70
Average number of items used	4.54	3.64	2
Correlation between thetas	0.98	0.96	0.95
Mean SE^a^	0.33	0.37	0.42
Item mean	4.54	3.64	2
Item median	3	2	2
Item SD^b^	3.36	3.34	0
Item range	2–12	2–12	2–2
Time of iterations	500	500	500

^a^SE = standard error.

^b^SD = standard deviation.

**Table 6:** Item information provided in specific range of full ENRICh.

Item	Specified range	Information provided for specified range (%)	Total information provided for the whole scale
All 15 items	(-10, +10)	78(100%)	78
All 15 items	(-2, +2)	70.32(90.15%)	78
Item 1	(-2, +2)	6.27(96.06%)	6.52
Item 2	(-2, +2)	5.35(93.23%)	5.74
Item 4	(-2, +2)	12.89 (99.52%)	12.95
Item 5	(-2, +2)	7.61 (96.55%)	7.88
Items1,2,4,5	(-2, +2)	32.11 (97.04%)	33.09

**Fig 6:**
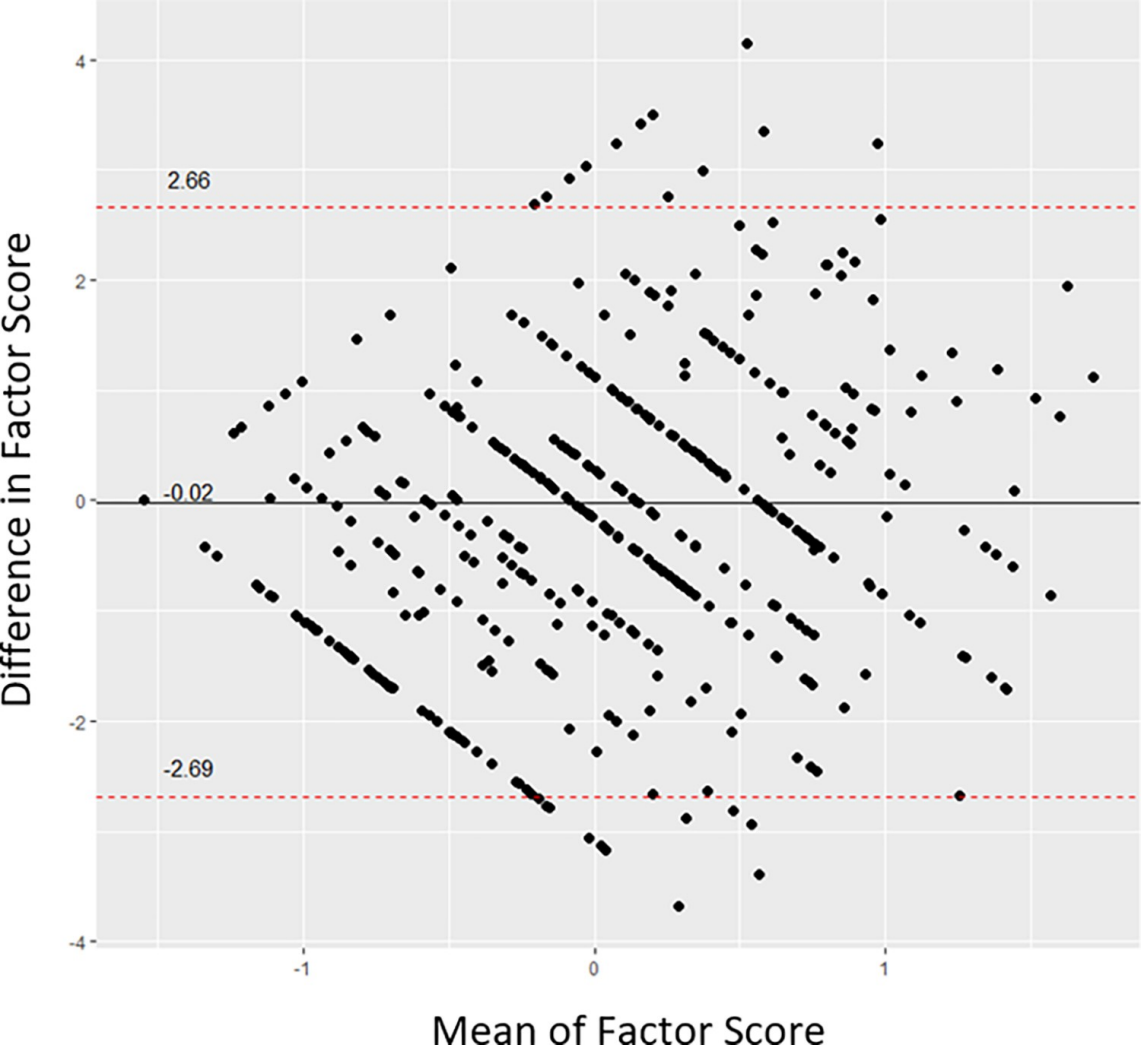
Bland-Altman plot of agreement between the CAT version and the fixed-length version with 12 items of ENRICh.

The agreement was evaluated between the CAT simulation and the fixed 12-item ENRICh measure using the Bland-Altman plot in Fig 6. The 95% limits of agreement ranged from -2.69 to +2.66 and only less than 5% of observations were outside this range.

### Comparison among full ENRICh, CAT, and ENRICh-4

**Fig 7:**
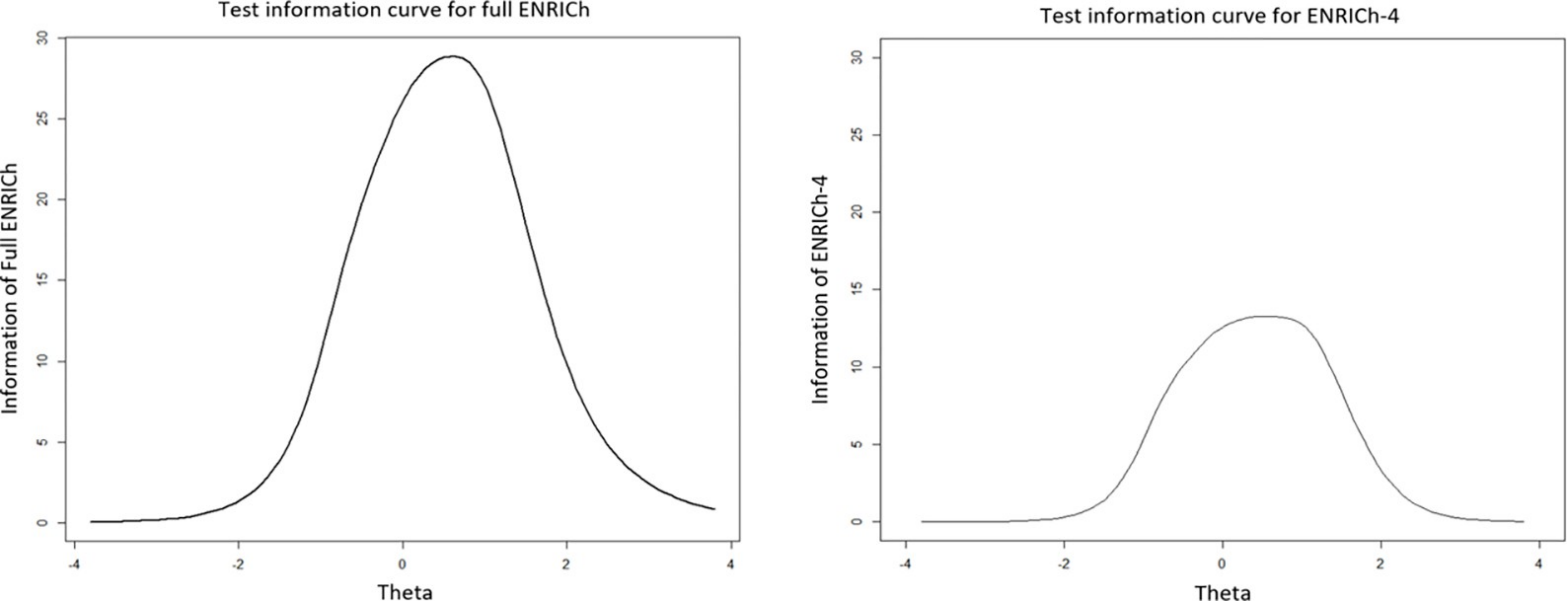
Test information curves for full ENRICh with 15 items and ENRICh with 4 items.

The basic information and comparison of participant scores among these three versions of ENRICh are displayed in Tables 7 and 8. The 4-item ENRICh was referred as ENRICh-4 version. The mean of participant scores for ENRICh-4 is highest (0.003, sd = 0.93); the root mean square deviation (RMSD = 0.31) of the participant scores between ENRICh-4 and ENRICh is largest. Fig 7 indicates that patients with higher-than-average levels of toxicity were most accurately measured using either the full ENRICh or ENRICh-4.

**Table 7:** Basic information of full ENRICh, CAT, and ENRICh-4.

Version	Included item (n)	Participant score				
		Mean	SD	Min	Max	Median
ENRICh	Items 1–15 (15)	-0.0004	0.96	-1.76	2.5	0.03
CAT^a^	Items 1–5,7–12,14 (12)	-0.045	0.92	-1.55	2.60	-0.08
ENRICh-4	Items 1,2,4,5 (4)	0.003	0.93	-1.30	1.94	0.02

^a^Results are from CAT 500 simulation with a stopping rule of SE = 0.32.

**Table 8:** Comparison of participant scores among full ENRICh, CAT, and ENRICh-4.

	Correlation between participant score	Mean difference	SD^a^ of difference	RMSD^b^
ENRICh-4 vs ENRICh	0.95	-0.003	0.31	0.31
CAT vs ENRICh^c^	0.98	-0.0002	0.19	0.19
ENRICh-4 vs ENRICh^c^	0.96	-0.0001	0.28	0.28

^a^SD = Standard deviation.

^b^RMSD = Root mean square deviation.

^c^ENRICh here excluded items 6,13,15 for CAT simulation.

## Discussion

### Main findings

Through advanced psychometric analysis of IRT, we developed a shorter version of the ENRICh measure as well as an efficient CAT version. Scores from both versions offer comparable scores to the full-length ENRICh. Applied in practice, these options are intended to reduce respondent burden and yet still provide an efficient means of identifying high risk patients needing intervention for financial toxicity. Simulated CAT version provides a novel option to improve efficiency and accuracy of PROM [17,29]. The 4-item short version provides an option to minimize administrative burden in settings where specific items to assess the broad range of dimensions of financial toxicity are not required. Moreover, the high reliability of this ENRICh measure (α = 0.92) derived from this American participant-focused study makes it more suitable to be utilized in the American clinical setting.

In this study, we explore a unidimensional factor structure for the ENRICh PROM, which has also been evaluated as a multidimensional measure [14]. While factor analytic methods only displayed moderate dimensionality, Mokken analysis demonstrated appropriate scaling along a single dimension.

In the item dependency assumption test, items 6 (ability to pay for food), 13 (having someone to help with your normal household responsibilities and daily chores), and 15 (having help from community resources) showed strong residual correlations. Many other plausible reasons could contribute to the above-mentioned distress, not closely associated with cancer treatment for patients. Therefore, they were all eliminated from further analyses due to lower information provide to this scale.

This study also ascertained eight uniform DIF items for age and race groups, which indicates that certain items are interpreted differently by different demographic groups [29]. Their uncrossing plots reflected that the demographic differences in these items are consistent along with the severity level of the financial toxicity continuum. Although the effect of the DIF items was not meaningful using the cut-off we had adopted for this study, we note that there 4 of 8 DIF items with beta change (β) were greater than the recommended cutoff point of 5% by other researchers [30].

During the CAT simulation, we note items 1 (money in your savings), 2 (money you owe), 4 (stress level about finances), and 5 (ability to pay bills) were the most frequently used. These items may be indicative of depleted coping resources and entering into a phase of increased financial toxicity, which is consistent with prior studies [24,31]. The strong correlation between factor score of items 1,2,4,5 and that of fixed 12-item scale makes the use of ENRICh-4 version possible. This ultra-short ENRICh version may provide a quick and convenient assessment of a unidimensional cancer patients’ financial burden for health care providers who prioritize brevity over assessment reliability in some circumstances. In addition, the ENRICh-4 version is well suited for screening purposes. For researchers and investigators who are interested in further understanding the dimensionality of financial toxicity, the full ENRICh is recommended.

Additionally, the available computer-adaptive measurement delivery platform—Concerto—has demonstrated the ability to move the transformative technology toward real clinical practice and research [32]. We will foresee that CAT implications will promote truly patient-centered care.

### Limitations

Some limitations in this study are summarized below. First, future research could generalize these results of the multi-institutional study to patients receiving care beyond the Houston metro area. Second, the precision estimation of an underlying trait in CAT simulation is slightly limited by the relatively small number of items of ENRICh [29]. Third, the performance of its application into different countries, languages, as well as the cross-cultural difference warrant further investigation. Fourth, the observed negative residual correlation among some items indicates the possibility of multidimensionality existence, suggesting the need for multidimensional CAT simulation to alleviate the controversy of the fairly weak dimensionality of ENRICh by incorporating additional information of items [33,34].

### Conclusion

This study shows that new short-form and adaptive versions of the ENRICh financial toxicity measure have acceptable psychometric properties, reduced redundancy, and simplified item response options through performing advanced psychometric analysis. Without sacrificing precision, the CAT version of ENRICh overperformed its fixed-length version in terms of the number of item administrations. The developed CAT version and ultra-short version containing four items alone are efficient screens for the severity of potential financial toxicity experienced by cancer patients, and also promote timely guidance and intervention provided to targeted populations.

## Supporting information

S1 Table ENRICh. (DOCX)

S2 Table Basic demographic and clinical information for 515 included patients. (DOCX)

S3 Table Revised ENRICh. (DOCX)

S1 Text Detailed analysis processes for IRT and CAT. (DOCX)

S1 Data (CSV)
