# Supplementary material for: Short-form adaptive measure of financial toxicity from the Economic Strain and Resilience in Cancer (ENRICh) study: Derivation using modern psychometric techniques
Source: PLoS One. 2022 Aug 25;17(8):e0272804. doi: 10.1371/journal.pone.0272804 (PMC9409561; doi:10.1371/journal.pone.0272804)
Supplement: S1 Table — (DOCX) [file pone.0272804.s001.docx]

**S1 Table. Economic Strain and Resilience in Cancer (ENRICh)^a^.**

| ***People with cancer may feel their disease or treatment affects their financial well-being.*** | | | | | | | | | | | | |
| --- | --- | --- | --- | --- | --- | --- | --- | --- | --- | --- | --- | --- |
| PLEASE RATE: During the **past month**, how has your disease and/or treatment affected… | Item number in original scale | Not affected at all |  |  |  |  |  |  |  |  |  | Affected a great deal |
| ^b^1. Money in your savings | 2 | 0 | 1 | 2 | 3 | 4 | 5 | 6 | 7 | 8 | 9 | 10 |
| 2. Other money you owe (like debts and credit cards) | 3 | 0 | 1 | 2 | 3 | 4 | 5 | 6 | 7 | 8 | 9 | 10 |
| 3. Your spending on medical bills | 1 | 0 | 1 | 2 | 3 | 4 | 5 | 6 | 7 | 8 | 9 | 10 |
| 4.Your stress level about finances | 8 | 0 | 1 | 2 | 3 | 4 | 5 | 6 | 7 | 8 | 9 | 10 |
| 5. Your ability to pay all of your bills | 4 | 0 | 1 | 2 | 3 | 4 | 5 | 6 | 7 | 8 | 9 | 10 |
| 6. Your ability to pay for food | 5 | 0 | 1 | 2 | 3 | 4 | 5 | 6 | 7 | 8 | 9 | 10 |
| 7. Your ability to work your usual number of hours at your job | 6 | 0 | 1 | 2 | 3 | 4 | 5 | 6 | 7 | 8 | 9 | 10 |
| 8. Your ability to contribute to your normal household responsibilities and daily chores | 7 | 0 | 1 | 2 | 3 | 4 | 5 | 6 | 7 | 8 | 9 | 10 |
| ***People with cancer may rely on a variety of financial resources and support sources.*** | | | | | | | | | | | | |
| PLEASE RATE: During the **past month**, to deal with the financial impact of your disease and/or treatment, how much did you rely on… | Item number in original scale | Did not rely at all |  |  |  |  |  |  |  |  |  | Relied a great deal |
| 9. Using your household income | 1 | 0 | 1 | 2 | 3 | 4 | 5 | 6 | 7 | 8 | 9 | 10 |
| 10. Using your savings | 2 | 0 | 1 | 2 | 3 | 4 | 5 | 6 | 7 | 8 | 9 | 10 |
| 11.Using credit cards | 3 | 0 | 1 | 2 | 3 | 4 | 5 | 6 | 7 | 8 | 9 | 10 |
| 12. Having someone to help manage your medical bills | 4 | 0 | 1 | 2 | 3 | 4 | 5 | 6 | 7 | 8 | 9 | 10 |
| 13. Having someone to help with your normal household responsibilities and daily chores | 5 | 0 | 1 | 2 | 3 | 4 | 5 | 6 | 7 | 8 | 9 | 10 |
| 14. Having someone to help care for the people who normally depend on you | 6 | 0 | 1 | 2 | 3 | 4 | 5 | 6 | 7 | 8 | 9 | 10 |
| 15. Having help from community resources (like churches, foundations, patient assistance, etc.) | 7 | 0 | 1 | 2 | 3 | 4 | 5 | 6 | 7 | 8 | 9 | 10 |

^a^ The Economic Strain and Resilience in Cancer (ENRICh) measure is adopted(1) .Its copyright is held by, and used with permission of, The University of Texas MD Anderson.

^b^ There are IRT item numbers. Items are re-ordered to all sub scales to be presented together for the convenience of analysis. Items can be presented in either the original or subscale format.

**Reference**

1. Smith GL, Mendoza TR, Lowenstein LM, Shih YCT. Financial hardship in survivorship care delivery. J Natl Cancer Inst - Monogr. 2021;2021(57):10–14. doi:10.1093/jncimonographs/lgaa012
