# Supplementary material for: Short-form adaptive measure of financial toxicity from the Economic Strain and Resilience in Cancer (ENRICh) study: Derivation using modern psychometric techniques
Source: PLoS One. 2022 Aug 25;17(8):e0272804. doi: 10.1371/journal.pone.0272804 (PMC9409561; doi:10.1371/journal.pone.0272804)
Supplement: S2 Table — (DOCX) [file pone.0272804.s002.docx]

**S2 Table. Basic demographic and clinical information for 515 included patients.**

| **Variable** | **Code** | **Value** | **Frequency**  **(%)** | **Missing N (%)** |
| --- | --- | --- | --- | --- |
| **Demographic information** | | | | |
| Age group |  | | | |
|  | 0 | Younger adults (<65) | 346(67%) | 0 |
|  | 1 | Old adults (>=65) | 169(33%) |  |
| Gender |  | | | |
|  | 0 | Female | 278(54%) | 0 |
|  | 1 | Male | 237(46%) |  |
| Race |  | | | |
|  | 0 | Non-white | 180(35%) | 0 |
|  | 1 | White | 335(65%) |  |
| **Socioeconomic information** | | | | |
| Education level |  | | | |
|  | 1 | Less than high school | 20(4%) | 4(1%) |
|  | 2 | High school or GED | 96(19%) |  |
|  | 3 | Some college, associate's degree, or trade certification | 174(34%) |  |
|  | 4 | College degree (BS, BA) | 133(26%) |  |
|  | 5 | Graduate degree (MS, MA) | 60(12%) |  |
|  | 6 | Advanced degree (PhD, MD, JD) | 28(5%) |  |
| Household income |  | | | |
|  | 1 | $0 to $9,999 | 42(8%) | 17(3%) |
|  | 2 | $10,000 to $14,999 | 16(3%) |  |
|  | 3 | $15,000 to $19,999 | 17(3%) |  |
|  | 4 | $20,000 to $34,999 | 46(9%) |  |
|  | 5 | $35,000 to $49,999 | 37(7%) |  |
|  | 6 | $50,000 to $74,999 | 69(13%) |  |
|  | 7 | $75,000 to $99,999 | 77(15%) |  |
|  | 8 | $100,000 to $199,999 | 110(21%) |  |
|  | 9 | $200,000 or more | 84(16%) |  |
| Working for pay |  | | | |
|  | 0 | No | 287(56%) | 6(1%) |
|  | 1 | Yes | 222(43%) |  |
| Employment status |  | | | |
|  | 0 | Unemployed | 282(55%) | 4(1%) |
|  | 1 | Employed | 229(44%) |  |
| ( **S2 Table** Continued) | | | | |
| **Variable** | **Code** | **Value** | **Frequency**  **(%)** | **Missing N (%)** |
| Number of types of insurance |  | | | |
|  | 0 | No | 7(1%) | 3(1%) |
|  | 1 | More than 1 types | 505(98%) |  |
| Marital status |  | | | |
|  | 1 | Married | 347(67%) | 6(1%) |
|  | 2 | Widowed | 22(4%) |  |
|  | 3 | Living as married | 9(2%) |  |
|  | 4 | Separated | 9(2%) |  |
|  | 5 | Divorced | 70(14%) |  |
|  | 6 | Single, never been married | 52(10%) |  |
| **Clinical information** | | | | |
| Diagnosis site |  | | | |
|  | 0 | Brain and other CNS | 4(1%) | 8(2%) |
|  | 1 | Breast | 211(41%) |  |
|  | 2 | Colorectal/anus | 22(4%) |  |
|  | 3 | GYN | 7(1%) |  |
|  | 4 | Head and neck | 29(6%) |  |
|  | 5 | Leukemia/Lymphoma/Myeloma | 23(4%) |  |
|  | 6 | Lung/Bronchus | 32(6%) |  |
|  | 7 | Neuroendocrine | 1(0%) |  |
|  | 8 | Other GI | 20(4%) |  |
|  | 9 | Other GU | 6(1%) |  |
|  | 10 | Prostate | 134(26%) |  |
|  | 11 | Skin | 5(1%) |  |
|  | 12 | Soft tissue | 7(1%) |  |
|  | 13 | Thymus | 2(0%) |  |
|  | 14 | Thyroid | 1(0%) |  |
|  | 15 | Unknown primary | 3(1%) |  |
| Diagnosis stage |  | | | |
|  | 0 | Acute | 4(1%) | 8(2%) |
|  | 1 | Distant | 90(17%) |  |
|  | 2 | Grade IV | 3(1%) |  |
|  | 3 | Local | 243(47%) |  |
|  | 4 | Myeloma | 5(1%) |  |
|  | 5 | Regional | 162(31%) |  |
