# Supplementary material for: Short-form adaptive measure of financial toxicity from the Economic Strain and Resilience in Cancer (ENRICh) study: Derivation using modern psychometric techniques
Source: PLoS One. 2022 Aug 25;17(8):e0272804. doi: 10.1371/journal.pone.0272804 (PMC9409561; doi:10.1371/journal.pone.0272804)
Supplement: S3 Table — (DOCX) [file pone.0272804.s003.docx]

**S3 Table. Revised Economic Strain and Resilience in Cancer (ENRICh)^a^.**

| ***People with cancer may feel their disease or treatment affects their financial well-being****.* | | | | |
| --- | --- | --- | --- | --- |
| PLEASE RATE: During the **past month**, how has your disease and/or treatment affected… | Item number in original scale | Not affected at all |  | Affected a great deal |
| 1.Your spending on medical bills | 1 | 0 | 1 | 2 |
| 2.Money in your savings | 2 | 0 | 1 | 2 |
| 3.Other money you owe (like debts and credit cards) | 3 | 0 | 1 | 2 |
| 4.Your ability to pay all of your bills | 4 | 0 | 1 | 2 |
| 5.Your ability to work your usual number of hours at your job | 6 | 0 | 1 | 2 |
| 6.Your ability to contribute to your normal household responsibilities and daily chores | 7 | 0 | 1 | 2 |
| 7.Your stress level about finances | 8 | 0 | 1 | 2 |
| ***People with cancer may rely on a variety of financial resources and support sources.*** | | | | |
| PLEASE RATE: During the **past month**, to deal with the financial impact of your disease and/or treatment, how much did you rely on… | Item number in original scale | Did not rely at all |  | Relied a great deal |
| 1.Using your household income | 1 | 0 | 1 | 2 |
| 2.Using your savings | 2 | 0 | 1 | 2 |
| 3.Using credit cards | 3 | 0 | 1 | 2 |
| 4.Having someone to help manage your medical bills | 4 | 0 | 1 | 2 |
| 5.Having someone to help care for the people who normally depend on you | 6 | 0 | 1 | 2 |

^a^ The Economic Strain and Resilience in Cancer (ENRICh) measure is adopted (1). Its copyright is held by, and used with permission of, The University of Texas MD Anderson.

**Reference**

1. Smith GL, Mendoza TR, Lowenstein LM, Shih YCT. Financial hardship in survivorship care delivery. J Natl Cancer Inst - Monogr. 2021;2021(57):10–14. doi:10.1093/jncimonographs/lgaa012
