# Supplementary material for: Short-form adaptive measure of financial toxicity from the Economic Strain and Resilience in Cancer (ENRICh) study: Derivation using modern psychometric techniques
Source: PLoS One. 2022 Aug 25;17(8):e0272804. doi: 10.1371/journal.pone.0272804 (PMC9409561; doi:10.1371/journal.pone.0272804)
Supplement: S1 Text — (DOCX) [file pone.0272804.s004.docx]

**Detailed Analysis Processes for IRT and CAT**

**Item response theory analysis**

We evaluated the internal consistency of the ENRICh by calculating the Cronbach's α; we then conducted the confirmatory factor analysis (CFA) to examine its predefined scale structure. If the original factor structure was not sufficient, we could further perform exploratory factor analysis (EFA) with Monte Carlo comparison and oblimin rotation to investigate the scale structure or use automated item selection procedure (AISP) of Mokken analysis as an alternative to verify our findings[1]. We adopted the following widely used statistics with recommend acceptable thresholds to evaluate the goodness of model fit: ꭓ^^2^(*p* >0.05), Tucker-Lewis index (TLI)≥0.90, Comparative fix index (CFI)≥0.90, root mean square error of approximation (RMSEA) <(.06-.08); root means square of the residual(RMSR)<(.06-.08)[2].

We then carried out the Mokken analysis to test the scalability[1].Both each item and whole scale with Loevinger's Ho value greater than 0.3 are considered to have sufficient scalability[3].Here each item of ENRICh scale has three response categories, Samejima's Graded response model (GRM), targeting polytomous data and more suitable for developing item bank for CAT[4,5], was employed to examine the item dependence issue, which was considered to exist when the Yen's Q3 of residual correlation between items larger than +0.2[6]. The violated items could be either removed from the scale on sound reason or place into a testlet[2].

Additionally, the applied GRM also yielded item parameters of discrimination denoted a and difficulty denoted b as thresholds, which illustrates the extent to which an item differentiates respondents on the underlying trait being measuring and the level of underlying trait that item is measuring, respectively. Wherein, disordered threshold issues may arise for some items and could be corrected by collapsing them into appropriate adjacent items according to their similar semantics, to ensure each response category of the items has a chance to be endorsed for questionnaire-takers[7].

We evaluated the differential item functioning (DIF) issue for age, gender, and race group, by applying the logistic regression procedure under the IRT framework[8], to account for the possibly appearing measurement variance in demographics[7]. The significance of the DIF item was set at the alpha value (α) of 0.01 in the likelihood ratio *R^2^* test[9]. Meaningful DIF items were considered with pseudo-R-squared greater 0.13 or beta change within the range of 5% to 10% in this study[8,10].These detected DIF items with unwanted bias may damage the test reliability and content validity[11]. The treatment of DIF items, depending on the purpose of that test[12],mainly consists of five distinct practices found after a review of 27 articles, of which, deleting and ignoring DIF items are dominant strategies[11].

All detected items with breach of assumption were excluded from scale completely, and the remaining items were reanalyzed to calibrate item parameters for subsequent CAT simulation.

We assessed the overall model fit of IRT GRM using M_2_ statistic[13].

**CAT simulation**

For CAT simulation in this study, we set the IRT scaling constant at 1.7; the first item with most information function to be administered by CAT was at distribution mean; the maximum posterior weighted information (MPWI) approach was adopted as an item selection criterion; SE calculation method was Posterior; this process of CAT would be terminated until pre-specified stopping rule has met, such as a time limit, certain number of items, or minimum standard error of measurement[14].Hence, the SEs of 0.32,0.45,0.55, equivalent to the reliability of 0.90,0.80,0.70, was set as a stopping rule to perform 3 times CAT simulation, respectively; the Bayesian expected a posteriori (EAP) theta estimator was used to estimate factor scores, which then were randomly resampled for 500 times. The response set was generated for each resampled factor score. The CAT algorithm attempted to reproduce resampled factor scores from plausible response sets. The criteria set has widely used and achieved excellent measurement performance for CAT simulation with polytomous items in our previous studies[2,3,15,16]. The agreement between factor score derived from the CAT simulation and the full scale were assessed by using 95% limits of agreement displayed in Bland-Altman Plot[17].

**References**

1. Sijtsma K, Meijer RR, Andries van der Ark L. Mokken scale analysis as time goes by: An update for scaling practitioners. Pers Individ Dif. 2011;50(1):31–37. doi:10.1016/j.paid.2010.08.016

2. Loe BS, Stillwell D, Gibbons C. Computerized adaptive testing provides reliable and efficient depression measurement using the CES-D scale. J Med Internet Res. 2017;19(9). doi:10.2196/jmir.7453

3. Gibbons C, Bower P, Lovell K, Valderas J, Skevington S. Electronic quality of life assessment using computer-adaptive testing. Journal of Medical Internet Research. 2016;18(9). doi:10.2196/JMIR.6053

4. Samejima F. Estimation of latent ability using a response pattern of graded scores. ETS Res Bull Ser. 1968;1968(1):i-169. doi:10.1002/j.2333-8504.1968.tb00153.x

5. Forero CG, Maydeu-Olivares A. Estimation of IRT graded response models: Limited versus full information methods. Psychol Methods. 2009;14(3):275-299. doi:10.1037/a0015825

6. Yen WM. Scaling performance assessments: Strategies for managing local item dependence. J Educ Meas. 1993;30(3):187–213. doi:10.1111/j.1745-3984.1993.tb00423.x

7. Bee P, Gibbons C, Callaghan P, Fraser C, Lovell K. Evaluating and quantifying user and carer involvement in mental health care planning (EQUIP): Co-development of a new patient-reported outcome measure. PLoS One. 2016;11(3):e0149973. doi:10.1371/journal.pone.0149973

8. Choi SW, Gibbons LE, Crane PK. Iordif: An R package for detecting differential item functioning using iterative hybrid ordinal logistic regression/item response theory and monte carlo simulations. J Stat Softw. 2011;39(8):1-30. doi:10.18637/jss.v039.i08

9. Swaminathan H, Rogers HJ. Detecting differential Item functioning using logistic regression procedures. J Educ Meas. 1990;27(4):361-370. doi:10.1111/j.1745-3984.1990.tb00754.x

10. Crane PK, Gibbons LE, Jolley L, Van Belle G. Differential item functioning analysis with ordinal logistic regression techniques: DIFdetect and difwithpar. Med Care. 2006;44(11 suppl 3):S115-123. doi:10.1097/01.mlr.0000245183.28384.ed

11. Cho SJ, Suh Y, Lee WY. After differential item functioning is detected: IRT item calibration and scoring in the presence of DIF. Appl Psychol Meas. 2016;40(8):573–591. doi:10.1177/0146621616664304

12. Borsboom D. The attack of the psychometricians. Psychometrika. 2006;71(3):425-440. doi:10.1007/s11336-006-1447-6

13. Cai L, Hansen M. Limited-information goodness-of-fit testing of hierarchical item factor models. Br J Math Stat Psychol. 2013;66(2):245-276. doi:10.1111/j.2044-8317.2012.02050.x

14. Dudek FJ. The continuing misinterpretation of the standard error of measurement. Psychol Bull. 1979;86(2):335-337. doi:10.1037/0033-2909.86.2.335

15. Gibbons CJ, Skevington SM. Adjusting for cross-cultural differences in computer-adaptive tests of quality of life. Qual Life Res. 2018;27(4):1027-1039. doi:10.1007/s11136-017-1738-7

16. Xu C, Schaverien M V., Christensen JM, Sidey-Gibbons CJ. Efficient and precise Ultra-QuickDASH scale measuring lymphedema impact developed using computerized adaptive testing. Qual Life Res. 2021;(0123456789). doi:10.1007/s11136-021-02979-y

17. Bland JM, Altman DG. Statistical methods for assessing agreement between two methods of clinical measurement. Lancet.1986;1(8476):307-310. PMID: 2868172.
